# Arsenical Poisoning

**Published:** 1896-03

**Authors:** 


### Arsenical Poisoning.

"Arsenic has been found in the urine when the source from which it came was not discovered."

"To see if any arsenic could come from the temporary fillings used by dentists I have made twelve examinations of gutta-percha stoppings and oxyphosphate cements, finding arsenic in nearly all of them, writes William H. Rollins to the Boston *Medical and Surgical Journal.* "To confirm the experiments, I have sent a sample of the cement I most frequently use in filling teeth to Dr. Hills, of the Haverford Medical School, who found one part in twelve hundred of arsenic. As these fillings are constantly dissolving and wearing away in the mouth, minute amounts of arsenic must go into the system in this way."

The above is a clipping from an exchange, the statement contained in which, will be a surprise to every dentist. Few, if any, have ever thought of arsenic being found in gutta-percha or in oxyphosphate of zinc, as used for filling teeth. An analysis was recently made of five samples of oxyphosphate, in only one of which was there found the slightest trace of arsenic, and in the gutta-percha material not the slightest trace detected.

It is difficult to imagine how arsenic could be present in these materials, and especially in the gutta-percha fillings. There is of course, a possibility that a trace of arsenic might be present in the oxyphosphate having been conveyed there in the zinc from which the material is prepared, and even were it in a relatively considerable amount in the metallic zinc, it would almost certainly be driven off by the heat employed in producing the oxid of this metal. Even admitting the presence of the amount above indicated, no mischief would be likely to occur from it, for only that on the surface of the filling could, by any possibility, be taken up by the patient, and this would be so small that it is hardly conceivable that it could make any impression upon even those most susceptible to the influence of this agent. Arsenic is used for medical purposes in vastly larger quantities than would be possible, in this way, without even a trace of its injurious influence.—[Ed. Register.]

